# Cost-effectiveness of emicizumab for the treatment of hemophilia A: a systematic review

**DOI:** 10.3389/fpubh.2025.1658760

**Published:** 2025-10-07

**Authors:** Min Chen, Yunzhu Lin, Guoqian He, Liang Huang, Junyi Han, Jiaqi Ni

**Affiliations:** ^1^Department of Pharmacy/Evidence-Based Pharmacy Center, West China Second University Hospital, Sichuan University, Chengdu, China; ^2^Key Laboratory of Birth Defects and Related Diseases of Women and Children, Sichuan University, Ministry of Education, Chengdu, China; ^3^West China School of Pharmacy, Sichuan University, Chengdu, China; ^4^Department of Pediatric Hematology and Oncology, West China Second University Hospital, Sichuan University, Chengdu, China

**Keywords:** hemophilia A, emicizumab, cost-effectiveness, systematic review, pharmacoeconomic

## Abstract

**Background:**

Emicizumab, a bispecific factor IXa- and factor X-directed antibody indicated for routine prophylaxis of bleeding episodes in people with hemophilia A, can impose a significant financial burden. We conducted a systematic review to evaluate the reporting quality of existing pharmacoeconomic studies on emicizumab, and to synthesize its cost-effectiveness for hemophilia A treatment.

**Methods:**

Databases including PubMed, Embase, Cochrane Library, National Health Service Economic Evaluation Database, Health Technology Assessment, China National Knowledge Infrastructure, VIP China Science and Technology Journal database, and WanFang were searched for pharmacoeconomic studies on emicizumab. The general information, methods, and results of the retrieved studies were analyzed. The reporting quality of the studies was evaluated with the Consolidated Health Economic Evaluation Reporting Standards (CHEERS) 2022 checklist.

**Results:**

A total of 163 studies were retrieved, and 17 studies were further analyzed. Emicizumab was compared to bypassing agents (BPAs), recombinant factor VIII (rFVIII), recombinant factor VIII Fc fusion protein (rFVIIIFc), and gene therapy. The reporting quality of the studies is generally good with an average score of 79.64% (22.3/28) based on the CHEERS 2022 checklist. Current studies revealed that emicizumab prophylaxis was more cost-effective compared to BPAs in people with hemophilia A with inhibitors. However, its cost-effectiveness compared to rFVIII was unclear and varied across different countries. In addition, rFVIIIFc and valoctocogene roxaparvovec were more cost-effective than emicizumab for people with HA without inhibitors.

**Conclusion:**

Emicizumab prophylaxis was more cost-effective compared to BPAs in people with hemophilia A with inhibitors. Cost-effectiveness analyses with more accurate cost estimations of different countries should provide more convincing evidence for clinical decision-making.

**Systematic review registration:**

Identifier CRD 42023429349, https://www.crd.york.ac.uk/PROSPERO/view/CRD42023429349.

## 1 Background

Hemophilia A (HA) is an X-linked congenital bleeding disorder caused by the deficiency of coagulation factor VIII (FVIII). According to the World Federation of Hemophilia’s (WFH) 2023 annual global survey, approximately 218,800 people were diagnosed with hemophilia globally, with 80–85% classified as HA cases (1). One of the main goals of HA treatment is to prevent joint injury and bleeding, with FVIII replacement therapy serving as the cornerstone of treatment (2, 3). The WFH 2020 guideline emphasizes that regular replacement therapy (prophylaxis) with clotting factor concentrates or other hemostasis products, such as emicizumab, is the standard of care for all patients with severe HA to prevent bleeding and associated complications, particularly musculoskeletal damage (3). In addition, Principle 8 of the guideline highlights early initiation of prophylaxis (ideally before age 3) to alter the natural history of the disease (3). Although conventional FVIII preparations and extended half-life (EHL) FVIII products can effectively control bleeding, the inconvenience of frequent intravenous administration remains a significant challenge in HA management. Moreover, the development of inhibitory antibodies against FVIII is the major complication of replacement therapy, leading to partial or complete treatment resistance. FVIII replacement therapy is less effective for those with inhibitors due to neutralization of FVIII by these antibodies. Notably, up to one-third of people with severe HA receiving FVIII prophylaxis develop inhibitors, which lead to considerable morbidity from joint deformities and increased mortality (2). For those with FVIII inhibitors, prophylaxis with bypassing agents (BPAs) has emerged as an effective strategy to significantly reduce bleeding episodes (4). Currently available BPAs include activated prothrombin complex concentrates (aPCC) and recombinant activated factor VII (rFVIIa). The International Society on Thrombosis and Hemostasis (ISTH) clinical practice guideline recommends that for severe/moderate HA without inhibitors, standard prophylactic with FVIII concentrates (standard or extended half-life recombinant formulations) should be used to reduce bleeding risks compared to on-demand therapy. EHL products offer reduced treatment frequency, while low-dose FVIII prophylaxis (10 IU/kg twice weekly) is recommended in resource-limited settings where standard prophylaxis is inaccessible. Additionally, emicizumab is suggested as an alternative to FVIII therapy due to its subcutaneous administration and less frequent dosing (weekly/biweekly/every 4 weeks). In those with FVIII inhibitors, ISTH recommends emicizumab prophylaxis over BPAs such as rFVIIa or aPCC, citing potential cost-effectiveness and reduced treatment burden (5).

Emicizumab is a recombinant, humanized, bispecific monoclonal antibody that restores the endogenous coagulation pathway by bridging activated factor IXa (FIXa) and factor X (FX). Unlike FVIII replacement therapy, emicizumab lacks structural homology with FVIII, thereby eliminating the risk of FVIII inhibitor formation. Additionally, emicizumab can be injected subcutaneously with a long half-life, allows less frequent intravenous administrations, and increases patient mobility and quality of life. Emicizumab is the first approved non-factor therapy for routine prophylaxis of bleeding episodes in adult and pediatric population with HA (6). The recommended loading regimen involves a 3 mg/kg loading dose administered subcutaneously once weekly for the first 4 weeks, followed by maintenance doses of 1.5 mg/kg once weekly, or 3 mg/kg once biweekly, or 6 mg/kg once every 4 weeks (6, 7). Clinical trials HAVEN 1–4 showed that emicizumab prophylaxis revealed low bleeding rates in people with HA of all ages with/without FVIII inhibitors (8). HAVEN1 revealed that the annual bleeding rate (ABR) was 2.9 events (95% CI, 1.7–5.0) in the emicizumab prophylaxis group, compared to 23.3 events (95% CI, 12.3–43.9) in the group without prophylaxis, showed a significant difference of 87% in favor of emicizumab prophylaxis (*p* < 0.001). Compared to BPAs prophylaxis, emicizumab reduced the length of hospitalization days and the loss of work days. The average length of stay was 4.2 days in the BPAs group and 1.9 days in the emicizumab group (9). In addition, emicizumab prophylaxis resulted in a bleeding rate that was significantly lower by 79% than the rate with previous BPAs prophylaxis (*p* < 0.001). The most frequently reported adverse events were injection site reactions. No antidrug antibodies were detected (10). On the contrary, a matching-adjusted indirect comparison study comparing emicizumab with recombinant factor VIII Fc fusion protein (rFVIIIFc) found that emicizumab (administered every 4 weeks) demonstrated significantly lower proportions of patients achieving zero bleeds compared to rFVIIIFc (29.3% vs. 51.2%, *p* = 0.03) (11). However, no significant differences were observed between rFVIIIFc and weekly/biweekly emicizumab in terms of ABR or zero-bleed rates. Safety data indicated higher incidences of injection site reactions with emicizumab (20–32%) compared to rFVIIIFc (0%) (11). Moreover, real-world evidence analysis of 131 people with HA without inhibitors switching from FVIII to emicizumab showed no significant reduction in ABR (0.25 vs. 0.20, *p* = 0.4456), suggesting comparable efficacy between the two regimens (12). The results align with clinical trial findings demonstrating non-inferiority of emicizumab compared to FVIII prophylaxis in inhibitor-free populations (11, 12). Collectively, these findings establish emicizumab as a superior option for inhibitor-positive patients while maintaining equivalence to standard FVIII therapy in inhibitor-free individuals.

HA imposes substantial economic burdens due to both direct medical costs and indirect expenses, such as breakthrough bleed management (13). Emicizumab has demonstrated cost-reducing potential in HA treatment. The Australian societal analysis conducted by Brown et al. showed emicizumab reduced first-year total costs by 62.3% (Australian dollar, AUD 69.197 million/ United States dollar, USD 96.184 million) compared to conventional therapy in inhibitor-positive and severe/moderate inhibitor-free populations. This included decreases in FVIII product use (64.2%), BPA costs (92%), non-treatment direct costs (30.7%, AUD 3.771 million/USD 5.242 million), and indirect costs (19.1%, AUD 2.732 million/USD 3.797 million) (14). A real-world study enrolling 92 people with HA reported median total costs dropped from USD 176,720 to 128,099 (*p* = 0.04) after initiating emicizumab prophylaxis (15). Cost modeling of emicizumab indicated comparable costs to standard half-life (SHL) FVIII products and lower costs than EHL FVIII in people with HA without inhibitors (15). Cost-effectiveness analysis performed by Agboola et al. found emicizumab provided equivalent bleeding outcomes and quality-adjusted life years (QALYs) at lower costs compared to FVIII prophylaxis, establishing it as a cost-saving strategy (16). These findings highlight emicizumab’s economic advantages across diverse populations and healthcare settings.

However, the results of cost-effectiveness analysis may vary between studies due to regional price differences in emicizumab, FVIII, and BPAs, as well as the lack of direct head-to-head comparison studies (since these drugs are not bioequivalent). Furthermore, the long-term cost-effectiveness of emicizumab remains unclear. To address these limitations and fill the gap of lacking global synthesis of such evidence and standardized quality assessment in prior studies, this systematic review aims to answer two core research questions: (1) What is the reporting quality of existing emicizumab pharmacoeconomic studies? (2) Is emicizumab more cost-effective for HA treatment compared with BPAs, recombinant factor VIII (rFVIII), rFVIIIFc, and gene therapy, particularly across patient subgroups with/without FVIII inhibitors? It synthesizes indirect evidence across diverse regions to inform clinical drug selection in HA treatment amid limited direct comparative data, while also evaluating the quality of published pharmacoeconomic studies.

## 2 Methods

This systematic review was conducted following the criteria of the Preferred Reporting Items for Systematic Reviews and Meta-Analyses (PRISMA) statement, and it was registered in the International Prospective Register of Systematic Reviews (PROSPERO), CRD 42023429349.

### 2.1 Inclusion and exclusion criteria

Pharmacoeconomic studies of emicizumab for HA treatment were retrieved. Study types included cost-effectiveness analysis, cost-utility analysis, cost–benefit analysis, and cost-minimization analysis. The language of the literature is limited to Chinese and English. Reviews, conference abstracts, and those with unclear reporting of economic evaluation indicators or results were excluded.

### 2.2 Search strategy

The study group performed a comprehensive search in PubMed, Embase, Cochrane Library, National Health Service Economic Evaluation Database (NHSEED), Health Technology Assessment (HTA), China National Knowledge Infrastructure (CNKI), VIP China Science and Technology Journal database (VIP), and WanFang databases for pharmacoeconomic studies on emicizumab on January 15, 2024. An updated search was conducted on September 2, 2025. Keywords included emicizumab, prophylactic therapy, cost-effectiveness, cost-utility, cost–benefit, cost minimization, economic, cost analysis, and pharmacoeconomic. The search used a combination of subject headings and free text.

### 2.3 Study selection and data extraction

Two researchers (MC and JN) independently screened the retrieved literature based on inclusion and exclusion criteria, and in case of disagreement, the decision was made by a third researcher (YL). If necessary, the original author was contacted to obtain relevant information. For the included literature, data was extracted and processed using a Microsoft Excel worksheet. The extracted information included the publication status of the literature (title, publication time, first author), research overview (disease studied, intervention measures, control measures), and information on pharmacoeconomic evaluation (research perspective, research period, study design, clinical efficacy results, cost types, discount rate). Additionally, currency conversions were performed using annual average exchange rates from the World Bank Open Data (17) for the study publication year, and all monetary amounts were standardized to USD for reporting purposes.

### 2.4 Evaluation of reporting quality

The reporting quality of the studies was evaluated with the Consolidated Health Economic Evaluation Reporting Standards (CHEERS) 2022 checklist (18) developed by the International Society for Pharmacoeconomics and Outcomes Research (ISPOR). The evaluation content involves 7 sections including title, abstract, introduction, methods, results, discussion, and other relevant information with a total of 28 items.

### 2.5 Data synthesis

Economic findings were synthesized and presented as a narrative summary in conjunction with a tabular summary. Given that there is high heterogeneity in terms of population, intervention, comparator, and outcome as well as economic evaluation frameworks across studies, a meta-analysis was not conducted. Instead, the three-by-three dominance ranking matrix (DRM) tool was adopted according to the systematic review of economic evaluation guidelines developed by the Joanna Briggs Institute (19). In the dominance raking framework, color coding was used to indicate implications for decision-makers. A red coding shows the situation in which a decision is less favored or rejected by decision-makers (the intervention is less effective with higher costs). A green coding indicates the case in which the intervention is strongly favored (the intervention is more effective with lower costs). A yellow coding shows that no decision can be made directly as the intervention is more effective with more costs or less effective with less costs. In that case, some forms of financial or clinical trade-off are required. An investigation of the threshold when the intervention is more cost-effective will also support the decision-making process.

## 3 Results

### 3.1 Results of the literature search

**Figure 1:**
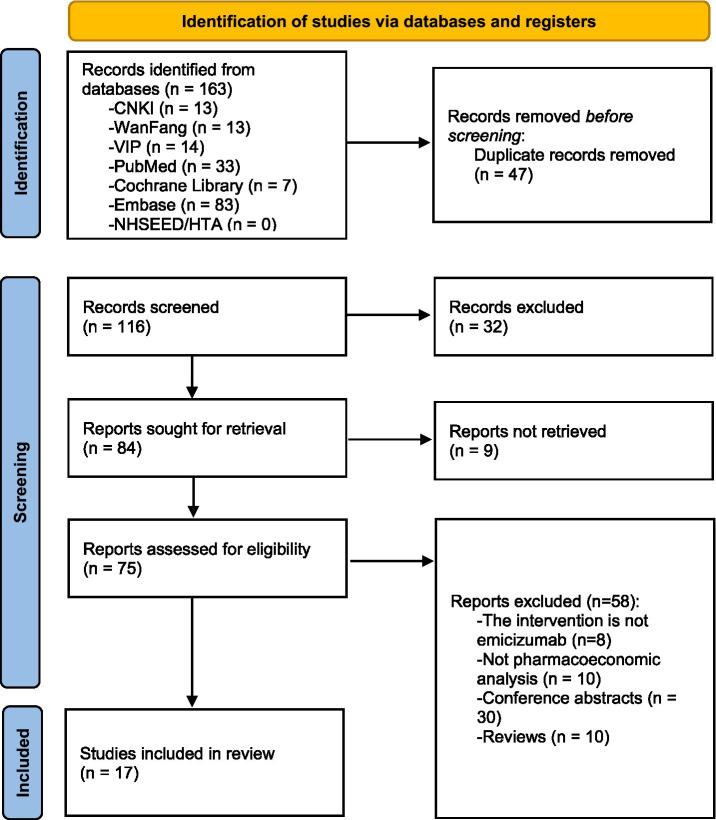
PRISMA flow diagram of literature search.

A total of 163 literature were retrieved, and 17 were ultimately included in the systematic review. The screening process of literature is shown in Figure 1.

### 3.2 General information of studies included

This study included a total of 17 articles published between 2020 and 2025 (16, 20–35). Among them, four studies were conducted in the United States, two in India, and other countries or regions included Iran, Thailand, Canada, Peru, Brazil, Italy, South Korea, France, the United Kingdom, Europe, and the Netherlands. Among them, eight articles conducted pharmacoeconomic studies on people with HA with inhibitors (20–27), and nine articles involved HA without inhibitors (16, 28–35). The types of pharmacoeconomic research included cost-effectiveness analysis, cost-utility analysis, and cost-minimization analysis. Eleven (64.71%) (16, 20–22, 24, 28–30, 32, 33, 35) articles conducted cost-effectiveness analysis, which is the most widely used type of economic research, using models such as Markov models, and decision tree model. The drugs compared with emicizumab included BPAs (aPCC and rFVIIa), recombinant factor VIII (rFVIII), rFVIIIFc, and valoctocogene roxaparvovec. The study perspectives included healthcare, payers, society, and patients. The study period covered 1.8 years to a lifetime, with 16 articles (94.12%) being long-term economic evaluations (lasted for 5 years or more) and 1 article (5.88%) (21) being short-term economic evaluations (lasted for less than 5 years). The most commonly used effectiveness indicators included ABRs and QALYs. The main cost type was the direct costs, including the costs of drug prophylaxis, and costs of managing bleeding and other adverse events. The general characteristics of the included studies are summarized in Table 1.

**Table 1:** General information about the studies included.

No	ID (author, year, Ref. no.)	Country	Population	Intervention	Comparator	Evaluation methods	Perspective	Cycle	Horizon	Cost types	Effectiveness	Discount	Economic results	Emicizumab more cost-effective? (Yes/No/Unclear)
People with HA with inhibitors														
1	Bitrán et al. (2022) (20)	Peru	Severe HA with inhibitors	Emicizumab	BPAs	Cost-effectiveness analysis with Markov model	Payer	1 week	Children: 16 years; adults: 52 years	Total treatment cost (drug, care, and bleeding management)	QALYs	3%	Emicizumab would generate savings between USD 14.6 million and USD 16.0 million per child and USD 11.8 million per adult.	Yes
2	Camelo et al. (2023) (21)	Brazil	Males aged 2 years with severe HA with inhibitors undergo immune tolerance induction	Emicizumab	BPAs (aPCC/ rFVIIa)	Cost-effectiveness analysis with decision tree model	Payer	N/A	1.8 years in the success group & 3.1 years in the failure group	Costs associated with the acquisition of the products and bleeding episode treatment	Total number of treated bleeding events	N/A	Immune tolerance induction with prophylaxis with BPAs resulted in an incremental cost of USD 724,478 and 8.65 bleeds per HA treatment compared with emicizumab	Yes
3	Cortesi et al. (2020) (22)	Italy	4-year-old population with HA with inhibitors	Emicizumab	BPAs	Cost-effectiveness analysis with Markov model	Payer	1 year	Lifetime	Treatment, major surgeries, and other direct costs	ABRs, QALYs	3%	Emicizumab was more effective (0.94 QALYs) and cost-saving (Euro 19.4 to 24.4 million/USD 22.89 to 28.79 million per patient compared with BPAs prophylaxis)	Yes
4	Lee et al. (2020) (23)	South Korea	HA with inhibitors	Emicizumab	BPAs	Cost-utility analysis with Markov model	Society	N/A	Lifetime	Costs of prophylaxis, bleeds & adverse events & transportation costs & caregiver’s costs	ABRs, QALYs	5%	Emicizumab prevented 807 bleedings, extended 3.04 QALYs, and reduced costs by USD 2.6 million	Yes
5	Polack et al. (2020) (24)	France	HA with inhibitors	Emicizumab	BPAs	Cost-effectiveness & cost-utility analysis with Markov model	Payer/society/patients	1 year	5 years	Prophylactic treatment, bleeding episodes, administration of drugs, adverse events, hospitalization and disease monitoring	ABRs, QALYs	4%	Emicizumab saved Euro 234,191/USD 276,345 for a gain of 0.88 QALYs	Yes
6	Saiyarsarai et al. (2021) (25)	Iran	HA with inhibitors	Emicizumab	BPAs (rFVIIa)	Cost-utility analysis with Markov model	Payer & society	1 year	Lifetime	Societal perspective: direct medical, direct non-medical & indirect expenses; Payer’s perspective: direct medical costs	QALYs	Cost: 5%; outcome:3%	The ICER for the group with ABR of 18 episodes per year at the age of 20 was USD 12,936 (<1–3 GDP)	Yes
7	Krishnamoorthy et al. (2024) (26)	India	HA with inhibitors	Emicizumab	BPAs (aPCC/rFVIIa)	Cost-utility analysis with Markov model	Health system	1 year	10 years	Costs of prophylaxis, bleeding events & severe adverse events	QALYs	3%	Emicizumab gain 7.18 QALYs while on-demand BPAs 6.45–6.55 QALYs. Prophylactic emicizumab was found to avert nearly 185 bleeding events against aPCC and 179 bleeding events against rFVIIa	Yes
8	Kengkla et al. (2024) (27)	Thailand	HA with inhibitors	Emicizumab	BPAs (aPCC/rFVIIa)	Cost-utility analysis with Markov model	Society	1 year	Lifetime	Direct medical costs (intervention, managing bleeding episodes, addressing arthroplasty complications, & hospitalization costs)	QALYs	3%	The ICER showed that emicizumab prophylaxis resulted in an incremental cost per QALY gained of −3,940,527 USD compared to BPAs prophylaxis and −729,851 USD compared to episodic treatment with BPAs.	Yes
People with HA without inhibitors														
9	Agboola et al. (2021) (16)	United States	HA without inhibitors	Emicizumab	Recombinant FVIII	Cost-effectiveness analysis with Markov model	Healthcare	6 months	Lifetime	Direct treatment costs associated with each regimen	QALYs, life year gained, and treated bleed avoided	3%	Emicizumab had lower costs with the same projected number of bleeds & QALYs vs. factor VIII (USD 13.598 million vs. USD 15.104 million)	Yes
10	Glaeser-khan et al. (2025) (28)	United States	Severe HA without inhibitors	Emicizumab	SHL FVIII	Cost-effectiveness analysis with Markov model	Society	1 month	Lifetime	Direct & indirect expenses	QALYs	3.5%	Emicizumab prophylaxis versus standard care accrued 25.6 and 25.1 QALYs across the lifespan at costs of 13.12 million and 13.07 million USD, respectively. The ICER for emicizumab was USD 99,900/QALY.	Yes
11	Ten Ham et al. (2022) (29)	Netherlands	Severe HA without inhibitors	Emicizumab	Valoctocogene roxaparvovec	Cost-effectiveness analysis with Markov model	Society	1 week	10 years	Healthcare and non-healthcare costs	ABRs, QALYs	Cost: 4.0%; QALYs: 1.5%	Valoctocogene roxaparvovec was more effective (0.13 QALYs) and cost-saving (Euro 1.41 million/USD 1.51 million) over a 10-year horizon.	No
12	Kragh et al. (2022) (30)	United Kingdom	Male population (≥12 years of age) with HA without inhibitors	Emicizumab	Recombinant factor VIII Fc	Cost-effectiveness analysis with Markov model	Not mentioned	6 months	Lifetime	Total treatment cost (prophylaxis treatment & bleeding management costs)	QALYs, total life years, number of bleeds	3.5%	rFVIIIFc was associated with lower costs (GBP 4.61 million/USD 5.85 million), a greater number of QALYs (0.014), and a lower number of bleeds (2.2)	No
13	Mancuso et al. (2022) (31)	Europe	HA without inhibitors	Emicizumab	Recombinant factor VIII Fc	Cost-minimization model	Payer	N/A	5 years	Costs of prophylaxis and breakthrough bleed treatment	N/A	N/A	Total incremental 5-year savings for rFVIIIFc rather than emicizumab use range from Euro 89,320,131/USD 113,436,566 to Euro 149,990,408/USD 190,487,818 in adolescents/adults (≥12 years) & Euro 173,417,486/USD 220,240,207 to Euro 253,240,465/USD 321,615,391 in children (<12 years)	No
14	Potnis et al. (2023) (32)	United States	Moderate or mild HA without inhibitors	Emicizumab	Recombinant FVIII	Cost-effectiveness analysis with Markov model	Payer	1 month	Lifetime	Costs of prophylaxis, bleeds & adverse events	QALYs	3%	Emicizumab extended 0.4 QALYs and raised costs by USD 5.8 million, ICER USD 14.5 million /QALY (>3 GDP)	No
15	Seth et al. (2024) (33)	India	HA without inhibitors	Emicizumab	Recombinant FVIII	Cost-effectiveness analysis with Markov model	Payers, patients & society	1 month	Lifetime	Direct & indirect expenses	QALYs	3.5%	Compared with high dose FVIII, the ICER/QALY was INR 27,869/USD 334.43. Emicizumab was considered a cost-effective option if the paying threshold was>1 GDP	Unclear
16	Yu et al. (2022) (34)	Canada	2-year-old male population with severe HA without inhibitors	Emicizumab	SHL FVIII, EHL FVIII	Cost-utility analysis with Markov model	Payer	1 month	Lifetime	Direct medical costs	QALYs	1.5%	Total cost per person for SHL FVIII, EHL FVIII, and emicizumab was USD 27.2 million, USD 36.7 million, and USD 26.2 million; Utility: 31.30, 31.16, and 31.61 QALYs, respectively.	Yes
17	Zhou et al. (2020) (35)	United States	Male children with severe HA without inhibitors	Emicizumab	SHL recombinant FVIII	Cost-effectiveness analysis with Markov model	Payer/society	1 week	Lifetime	Direct costs, indirect costs	ABRs, the mean ages at FVIII inhibitor development & arthropathy onset	3%	The cumulative number of all treated bleeds and joint bleeds avoided on emicizumab versus FVIII prophylaxis were 278.2 and 151.7, respectively. Emicizumab saved USD 7.58 million over a lifetime.	Yes

^*^HA, hemophilia A; QALYs, quality-adjusted life years; ICER, incremental cost-effectiveness ratio; ABRs, annual bleeding rates; FVIII, factor VIII; SHL FVIII, standard half-life factor VIII; EHL FVIII, extended half-life factor VIII; BPAs, bypassing agents; aPCC, activated prothrombin complex concentrate; rFVIIa, recombinant activated factor VII. Currency conversions were performed using annual average exchange rates from the World Bank Open Data (17) for the study publication year. Rates applied: 1 Euro = 1.18 USD (year 2020), 1 Euro = 1.07 USD (year 2022), 1 GBP = 1.27 USD (year 2022), 1 INR = 0.012 USD (year 2023).

### 3.3 Quality assessment of literature included

Evaluated by the CHEERS 2022 checklist (18), the reporting quality of the studies included was generally good, with an average score of 79.64% (22.3/28). The detailed grading scores are demonstrated in Table 2. 76.47% (13/17) of the studies did not describe any approaches to engage patients or service recipients, the general public, communities, or stakeholders in the study’s design. 58.82% (10/17) of studies did not describe any methods used for estimating how the results of the study varied for subgroup. 47.06% (8/17) of the studies did not provide relevant contextual information that may influence findings. 41.18% (7/17) of the studies did not describe how impacts were distributed across different individuals or adjustments made to reflect priority populations.

**Table 2:** Literature reporting quality based on CHEERS checklist.

ID (author, year, Ref. no.)	1	2	3	4	5	6	7	8	9	10	11	12	13	14	15	16	17	18	19	20	21	22	23	24	25	26	27	28	Scores
Agboola et al. (2021) (16)	√	√	√	√	√	√	√	√	√	√	√	√	√	√	x	√	√	√	√	√	√	√	√	√	√	√	√	√	27
Bitrán et al. (2022) (20)	√	√	√	√	√	x	√	√	√	√	√	√	√	√	√	√	√	x	√	√	x	√	√	x	x	√	√	√	23
Camelo et al. (2023) (21)	√	√	√	√	√	√	√	√	√	x	√	√	√	√	√	√	√	x	−	√	x	√	x	√	x	√	√	√	22
Cortesi et al. (2020) (22)	√	√	√	√	√	x	√	√	√	√	√	x	√	√	√	√	√	x	x	√	x	√	√	√	√	√	√	√	23
Glaeser-khan et al. (2025) (28)	√	√	√	√	√	√	√	√	√	√	√	√	√	√	√	√	√	x	−	√	x	√	√	√	x	√	√	√	23
Ten Ham et al. (2022) (29)	√	√	√	√	√	x	√	√	√	x	√	√	√	√	√	√	√	x	x	√	x	√	x	√	x	√	√	√	21
Kengkla et al. (2024) (27)	√	√	√	√	−	√	√	√	√	√	√	√	√	√	√	√	√	x	−	√	x	√	√	√	√	√	√	√	24
Kragh et al. (2022) (30)	√	√	x	√	√	x	√	x	√	x	√	x	√	√	√	√	√	√	√	x	√	√	x	√	√	√	√	√	21
Krishnamoorthy et al. (2024) (26)	√	√	√	√	√	√	√	√	√	√	√	√	√	x	x	√	√	x	−	√	x	√	√	√	√	√	√	√	23
Lee et al. (2020) (23)	√	√	√	√	√	x	√	√	√	√	√	√	√	√	√	√	√	x	x	x	x	x	√	√	√	√	x	√	21
Mancuso et al. (2022) (31)	√	√	√	√	√	x	√	√	√	√	−	−	√	√	x	√	√	√	√	−	−	x	−	√	√	√	x	√	19
Polack et al. (2020) (24)	√	√	x	√	√	√	√	x	√	−	−	√	√	√	√	√	√	√	√	√	√	√	√	√	√	√	x	√	23
Potnis et al. (2023) (32)	√	√	√	√	√	√	√	√	√	√	√	√	x	√	√	√	√	x	x	√	x	√	x	√	√	√	√	√	23
Saiyarsarai et al. (2021) (25)	√	√	√	√	√	x	√	√	√	√	−	√	√	√	x	√	√	√	x	√	x	√	√	√	x	√	x	x	20
Seth et al. (2024) (33)	√	√	√	√	√	x	√	√	√	√	x	√	x	√	x	√	√	x	x	√	x	√	x	√	x	√	√	√	19
Yu et al. (2022) (34)	√	√	√	√	√	√	√	√	√	√	√	√	√	√	√	√	√	√	x	√	x	√	−	√	-	√	−	√	23
Zhou et al. (2020) (35)	√	√	√	√	−	√	√	√	√	√	√	√	√	√	√	√	√	√	√	x	x	√	x	√	√	√	x	√	24

^*^√: meet the requirement; x: do not meet the requirement; −: not mentioned in the article; 1. Identify the study as an economic evaluation and specify the interventions being compared. 2. Provide a structured summary that highlights context, key methods, results, and alternative analyses. 3. Give the context for the study, the study question, and its practical relevance for decision-making in policy or practice. 4. Indicate whether a health economic analysis plan was developed and where available. 5. Describe characteristics of the study population (such as age range, demographics, socioeconomic, or clinical characteristics). 6. Provide relevant contextual information that may influence findings. 7. Describe the interventions or strategies being compared and why chosen. 8. State the perspective(s) adopted by the study and why chosen. 9. State the time horizon for the study and why appropriate. 10. Report the discount rate(s) and reason chosen. 11. Describe what outcomes were used as the measure(s) of benefit(s) and harm(s). 12. Describe how outcomes used to capture benefit(s) and harm(s) were measured. 13. Describe the population and methods used to measure and value outcomes. 14. Describe how costs were valued. 15. Report the dates of the estimated resource quantities and unit costs, plus the currency and year of conversion. 16. If modeling is used, describe in detail why used. Report if the model is publicly available and where it can be accessed. 17. Describe any methods for analyzing or statistically transforming data, any extrapolation methods, and approaches for validating any model used. 18. Describe any methods used for estimating how the results of the study vary for subgroups. 19. Describe how impacts are distributed across different individuals or adjustments made to reflect priority populations. 20. Describe methods to characterize any sources of uncertainty in the analysis. 21. Describe any approaches to engage patients or service recipients, the general public, communities, or stakeholders (such as clinicians or payers) in the design of the study 22. Report all analytic inputs (such as values, ranges, and references) including uncertainty or distributional assumptions. 23. Report the mean values for the main categories of costs and outcomes of interest and summarize them in the most appropriate overall measure. 24. Describe how uncertainty about analytic judgments, inputs, or projections affects findings. Report the effect of the choice of discount rate and time horizon, if applicable. 25. Report on any difference between patient/service recipient, general public, community, or stakeholder involvement made to the approach or findings of the study. 26. Report key findings, limitations, ethical or equity considerations not captured, and how these could affect patients, policy, or practice. 27. Describe how the study was funded and any role of the funder in the identification, design, conduct, and reporting of the analysis 28. Report authors’ conflicts of interest according to journal or International Committee of Medical Journal Editors requirements.

### 3.4 Economic evaluation results of the literature included

#### 3.4.1 Emicizumab versus BPAs

Eight studies (20–27) compared the cost-effectiveness of emicizumab and BPAs prophylaxis for people with HA with inhibitors. Compared to BPAs, overall, emicizumab prophylaxis is a more cost-effective option.

From the payer’s perspective, Bitrán et al. (20) conducted a cost-effectiveness analysis of emicizumab compared to BPAs with the Markov model for people with severe HA with inhibitors in Peru. The total treatment cost including the costs of drug, care, and bleeding management was estimated. The study revealed that compared to the current BPAs prophylaxis, emicizumab saved USD 14.6 to 16.0 million per child and USD 11.8 million per adult in the Ministry of Health. Social Security Health Insurance savings would be USD 12.8 to 14.9 million per child and USD 40.1 million per adult. The budget impact would be a net annual saving of USD 12.8 and 15.0 million in those entities. QALYs would be increased by 0.36 per child and 0.56 per adult, and 0.25 per child and 0.36 per adult in those respective institutions. In conclusion, emicizumab is a dominant strategy since it increases effectiveness and reduces costs compared to the current BPAs prophylaxis in people with severe HA with inhibitors. Camelo et al. (21) conducted a cost-effectiveness analysis with a decision tree model from the perspective of Brazilian government payers on the use of emicizumab and BPAs for males aged 2 years with severe HA and high-responding inhibitors undergoing immune tolerance induction (ITI). Costs associated with the acquisition of the drug and bleeding episode treatment were estimated. The results revealed that ITI with prophylaxis with BPAs resulted in an incremental cost of USD 724,478 and 8.65 bleeds per HA treatment compared with emicizumab, which supported the cost-effectiveness of emicizumab prophylaxis. Cortesi et al. (22) found that from the perspective of the Italian National Health Service (NHS), in population aged 4 years old with HA with inhibitors who failed ITI, emicizumab prophylaxis was found to be more effective (0.94 QALYs) and cost saving (European dollar, Euro 19.4 to 24.4 million/USD 22.89 to 28.79 million per patient lifetime) compared to BPAs prophylaxis. The cost-utility analysis performed by Lee et al. (23) revealed that emicizumab prophylaxis is more cost-effective compared to BPAs on-demand use in people with HA with inhibitors in South Korea. Lifetime emicizumab prophylaxis prevented 807 bleedings, extended 3.04 QALYs, and reduced costs by USD 2.6 million. In France, Polack et al. (24) found that in people with HA with inhibitors, emicizumab saved Euro 234,191/USD 276,345 for a gain of 0.88 QALYs. Saiyarsarai et al. (25) compared the economic benefits of the prophylactic use of emicizumab and the on-demand use of rFVIIa in people with HA with inhibitors from the perspectives of Iranian society and payers. The results demonstrated that emicizumab was cost-dominant compared to on-demand rFVIIa treatment across all age groups with an ABR of 20 to 25 episodes per year. The incremental cost-effectiveness ratio (ICER) for patients aged 20 years with an ABR of 18 episodes per year was calculated as USD 12,936 per QALY, which was lower than the 1–3 per capita gross domestic product (GDP) of Iran. Krishnamoorthy et al. (26) evaluated the cost-utility of emicizumab versus BPAs in the treatment of patients with severe HA with inhibitors in India. A Markov model was developed from the perspective of the health system, focusing on a hypothetical cohort of 10-year-old adolescents, with the analysis time horizon set at 10 years. Results revealed that prophylactic emicizumab therapy was a cost-saving intervention compared with both types of BPAs. The negative ICER was −853,573 USD relative to rFVIIa, and −211,675 USD relative to aPCC, respectively. Kengkla et al. (27) utilized a Markov model to estimate the cost-effectiveness of prophylactic emicizumab versus BPAs in HA patients with inhibitors in Thailand. From a societal perspective and with a lifetime time horizon adopted for the analysis, emicizumab prophylaxis resulted in higher QALYs at 61.56, compared to 43.50 for BPAs prophylaxis and 31.07 for episodic treatment with BPAs. The ICER showed that emicizumab prophylaxis resulted in an incremental cost per QALY gained of −3,940,527 USD compared to BPAs prophylaxis and −729,851 USD compared to episodic treatment with BPAs.

#### 3.4.2 Emicizumab versus rFVIII

Six studies (16, 28, 32–35) evaluated the cost-effectiveness of emicizumab and rFVIII prophylaxis for people with HA. The results differed between studies.

Four studies (16, 28, 34, 35) revealed that emicizumab was more cost-effective. From the United States (US) payer and societal perspective, the Institute for Clinical and Economic Review (the ICER, US research group) (16) compared the cost-effectiveness of emicizumab prophylaxis with rFVIII for people with HA without inhibitors. Emicizumab prophylaxis was found to have lower costs with the same projected number of bleeds and QALYs, with USD 1.506 million saved compared with rFVIII. Another study conducted by Glaeser-Khan et al. (28), which was carried out from the US societal perspective, aimed to evaluate the cost-effectiveness of emicizumab prophylaxis versus on-demand SHL FVIII for males with severe HA in their first year of life. Over the study participants’ lifespan, emicizumab prophylaxis and standard care (on-demand SHL FVIII) accrued 25.6 and 25.1 QALYs, respectively, with corresponding lifetime costs of USD 13.12 million and USD 13.07 million. The ICER of emicizumab versus standard care was USD 99,900 per QALY, which was lower than the willingness-to-pay threshold of USD 150,000 per QALY. In Canada, a study was conducted by Yu et al. (34), and emicizumab prophylaxis was found to be cost-saving among 2-year-old male population with severe HA, dominating the standard of care prophylaxis strategy. Emicizumab treatment resulted in 29 and 16 fewer bleeds in a lifetime compared to SHL FVIII and EHL FVIII, respectively. Total cost per person for SHL FVIII, EHL FVIII, and emicizumab was USD 27.2 million, USD 36.7 million, and USD 26.2 million, respectively; utility was 31.30, 31.16, and 31.61 QALYs, respectively. Zhou et al. (35) found that across all time assessment horizons, from US payer and societal perspective, emicizumab prophylaxis in male children with severe HA without any joint damage, the total costs were lower compared to SHL rFVIII. The respective total costs estimated for emicizumab versus rFVIII prophylaxis were USD 97,159 versus 331,610 at 1 year; USD 603,146 versus 1,459,496 at 5 years; and USD 15,238,072 versus 22,820,281 over a lifetime. In addition, emicizumab showed better clinical outcomes, including less reporting of bleeding events, delayed onset of arthropathy, and inhibitor development. The cumulative number of all treated bleeds and joint bleeds avoided on emicizumab versus FVIII prophylaxis were 278.2 and 151.7, respectively.

However, two studies (32, 33) revealed different results. Potnis et al. (32) compared the cost-effectiveness of emicizumab and FVIII in people with mild or moderate HA without inhibitors from a US payer’s perspective. Results of the cost-effectiveness analysis showed that emicizumab was more effective with 0.4 QALYs extended, however, it was more costly with USD 5.8 million raised. The ICER of emicizumab was USD 14.5 million per QALY (>3 GDP). From the perspective of Indian payers, patients, and society, Seth et al. (33) compared the cost-effectiveness of emicizumab prophylaxis (administered at a maintenance dose of 1.5 mg/kg weekly, 3 mg/kg every 2 weeks, or 6 mg/kg every 4 weeks) with FVIII-based regimens: on-demand therapy (ODT), low-dose prophylaxis (LDP, 1565 IU/kg/year), intermediate-dose prophylaxis (IDP, 3915 IU/kg/year), and high-dose prophylaxis (HDP, 7125 IU/kg/year). The results revealed that emicizumab was more cost-effective compared to HDP of FVIII-based regimens in people with HA without inhibitors, with an ICER of Indian rupees (INR) 27,869/USD 334.43 per QALY. Compared to IDP, ODT, and LDP, emicizumab prophylaxis could be considered a cost-effective option if the paying threshold is >1 per capita GDP.

#### 3.4.3 Emicizumab versus rFVIIIFc

Two studies (30, 31) evaluated the cost-effectiveness of emicizumab and rFVIIIFc prophylaxis for people with HA without inhibitors. Compared to rFVIIIFc, emicizumab was less cost-effective.

Kragh et al. (30) conducted a cost-effectiveness analysis of emicizumab and rFVIIIFc in male population (≥12 years of age) with HA without inhibitors in the United Kingdom (UK), which revealed that rFVIIIFc prophylaxis was more cost-effective compared to weekly emicizumab, with lower costs, a greater number of QALYs, and a lower number of bleeds. In 23.23 life years, the total costs of rFVIIIFc treatment were Great Britain pound (GBP) 5,978,424/USD 7,592,598 in comparison with GBP 10,593,306/USD 13,453,498 for emicizumab, total QALYs were 15.497 and 15.483, and the total discounted number of bleeds were 42.140 and 44.340, for rFVIIIFc and emicizumab, respectively. Mancuso et al. (31) established a cost-minimization model to compare emicizumab with rFVIIIFc when used in people with HA without inhibitors in Europe. They found rFVIIIFc was more cost-saving. Total incremental 5-year savings for rFVIIIFc rather than emicizumab use range from Euro 89,320,131/USD 113,436,566 to Euro 149,990,408/USD 190,487,818 in adolescents/adults (≥12 years), and Euro 173,417,486/USD 220,240,207 to Euro 253,240,465/USD 321,615,391 in children (<12 years). They discussed that the difference might be attributed not only to the high acquisition costs of emicizumab but also to additional expenses, such as drug wastage due to the mismatch between available vial sizes and weight-based dosing requirements.

#### 3.4.4 Emicizumab versus valoctocogene roxaparvovec

The emergence of gene therapies is the latest innovation in HA treatment (29). In June 2023, valoctocogene roxaparvovec, the first and only US Food and Drug Administration (FDA)-approved gene therapy for severe HA (congenital FVIII deficiency with FVIII activity < 1 IU/dL), was granted approval for adults without pre-existing antibodies to adeno-associated virus serotype 5 or FVIII. This one-time intravenous treatment uses an adeno-associated virus serotype 5 viral vector to deliver a transgene encoding coagulation FVIII, aiming to restore endogenous FVIII production and prevent bleeding events by correcting the mutant gene (36).

One study compared the cost-effectiveness of valoctocogene roxaparvovec with emicizumab. Ten Ham et al. (29) performed a cost-effective analysis with the Markov model to compare emicizumab with valoctocogene roxaparvovec from the perspective of Netherlands society. The results revealed that valoctocogene roxaparvovec resulted in improved health and lower cost compared to emicizumab in people with severe HA without inhibitors. Over a 10-year horizon, valoctocogene roxaparvovec was more effective and cost-saving compared to emicizumab (7.03 vs. 6.90 QALYs, Euro 2,839,210/USD 3,037,954 vs. Euro 4,252,167/USD 4,549,818). Valoctocogene roxaparvovec’s base case maximum value-based price (MVBP) was estimated at Euro 3.5 million/USD 3.75 million per treatment versus emicizumab. The mean break-even time was 5.68 years compared to emicizumab.

#### 3.4.5 Summary of the results of economic evaluations

**Figure 2:**
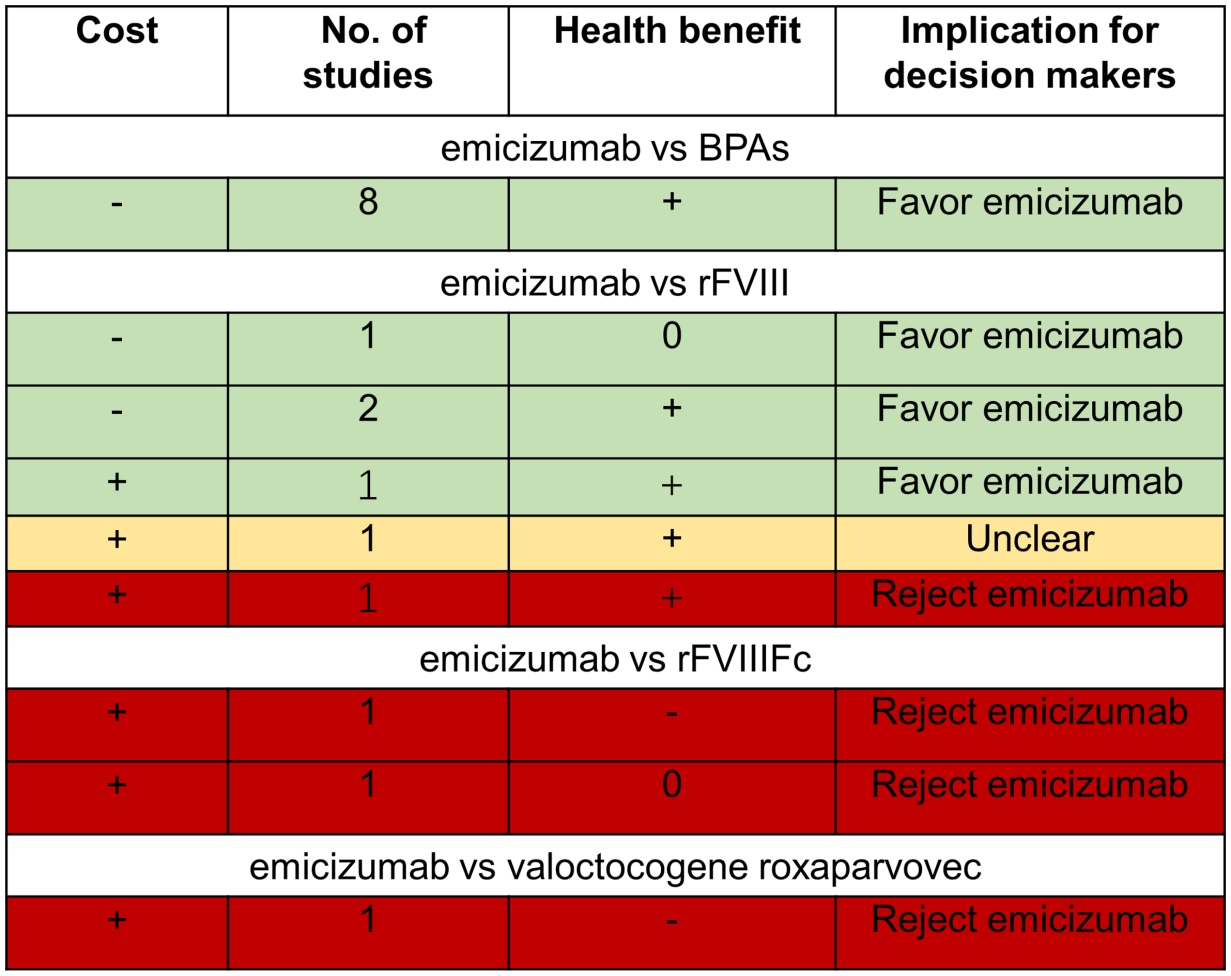
Three-by-three dominance ranking matrix. *+: emicizumab has a greater cost or greater health effect than the comparator; 0: emicizumab has equal cost or health effect/benefit as comparator; −: emicizumab is less costly or less effective than comparator. Read matrix by row left to right. BPAs: bypassing agents; rFVIII: recombinant factor VIII; rFVIIIFc: recombinant factor VIII Fc fusion protein.

Based on the above, the cost-effectiveness of emicizumab prophylaxis when compared with BPAs, FVIII, rFVIIIFc, and valoctocogene roxaparvovec is summarized in the DRM (Figure 2). As demonstrated in Figure 2, emicizumab prophylaxis is more cost-effective in people with HA with inhibitors compared with BPAs. Emicizumab prophylaxis is less cost-effective compared with rFVIII Fc and valoctocogene roxaparvovec in people with HA without inhibitors. The cost-effectiveness of emicizumab compared with rFVIII remains controversial. Whether emicizumab is more cost-effective or not needs to be determined based on the local per capita GDP level.

## 4 Discussion

Emicizumab is used to prevent bleeding in people with HA, which avoids the inconvenience of frequent intravenous injections, provides a new therapeutic drug choice for clinical practice, and improves the quality of life of patients. However, its high price makes the cost-effectiveness of emicizumab uncertain. To our knowledge, no systematic review on the pharmacoeconomic study of emicizumab has been published before. Most relevant studies focused on the cost-effectiveness of emicizumab compared with other treatment regimens in a certain country or region. In addition, some budget impact analysis was conducted. Watanabe et al. (37) established a budget impact model to preliminarily analyze the impact of emicizumab on the health budget in 5 years compared with no prevention regimen from the perspective of Malaysia’s public health system. The results showed that the introduction of emicizumab led to a 5-year cumulative incremental budget impact of USD 23,479,579 and a total of 72 participants received emicizumab. There was a cost reduction of USD 14,793,528 related to fewer hospitalizations and outpatient visits that required treatment with BPAs. The average cost per patient over the 5-year cumulative period was USD 115,979 when treated with emicizumab. The sensitivity analysis showed that if the ABR was greater than 16 times per year, emicizumab could save medical costs.

This study systematically retrieved the pharmacoeconomic studies of emicizumab, evaluated the reporting quality of each study, extracted the data, and summarized and interpreted the conclusions of each study. Based on the CHEERS 2022 checklist, the reporting quality of studies included was generally good, with an average score of 79.64% (22.3/28). Key quality gaps were identified: 76.47% of studies insufficiently described stakeholder engagement (e.g., patients or service recipients, the general public, communities, or stakeholders) in study design; 58.82% lacked methods for estimating how study results varied across subgroups; 47.06% failed to provide contextual information that may influence findings (e.g., regional healthcare policies, patient demographic characteristics, or local drug pricing); and 41.18% omitted descriptions of how impacts were distributed across individuals or adjustments made to reflect priority populations (e.g., pediatric, older adults, or resource-limited settings). These omissions collectively undermine study external validity: stakeholder input ensures alignment with real-world clinical needs, contextual information supports cross-region generalizability, and subgroup/distributional details enable targeted decision-making for diverse HA populations. Without these elements, findings may be less relevant to end-users (e.g., clinicians, payers, or health policymakers) and risk misinforming resource allocation for emicizumab in HA treatment.

This study retrospectively reviewed relevant literature and discussed the perspectives of analysis, analysis model, sensitivity analysis, model validation, and other factors affecting cost-effectiveness analysis. The aim is to summarize and interpret the results of the existing literature and provide reliable references for future research. Our study revealed that emicizumab prophylaxis was a more cost-effective option compared with BPAs in people with HA with inhibitors. Compared with rFVIII, results varied across studies. Compared with rFVIIIFc, emicizumab was a less cost-effective option for people with HA without inhibitors.

With the rapid advancement of gene therapy, the use of emicizumab and gene therapy for treating people with HA warrants further investigation. Our review identified a study demonstrating that valoctocogene roxaparvovec improved health outcomes and reduced costs compared to emicizumab prophylaxis in people with severe HA without inhibitors (29). Additionally, while not included in our final analysis due to predefined inclusion criteria, a notable study by the ICER (US research group) (38) employed decision-analytic models to compare valoctocogene roxaparvovec and emicizumab from a healthcare sector perspective over a lifetime horizon for adults with severe HA without inhibitors. Valoctocogene roxaparvovec demonstrated dominance in traditional full cost-offset analysis, projecting lower lifetime costs (USD 14.08 vs. 18.08 million for emicizumab) and marginally higher QALYs (17.57 vs. 17.47). However, when applying a USD 150,000 annual cap on cost offsets (a scenario deemed more policy-relevant due to the high baseline costs of factor therapies), the health benefit price benchmark for valoctocogene roxaparvovec was estimated at USD 1.958 to 1.963 million, significantly lower than its placeholder price of USD 2.5 million. The analysis highlighted substantial durability uncertainties, with FVIII levels declining by nearly 50% between 12- and 24-months post-treatment, and noted that no direct comparative evidence exists between valoctocogene roxaparvovec and emicizumab. The California Technology Assessment Forum (CTAF) panel voted unanimously that evidence was insufficient to distinguish net health benefits between the two therapies. Given the limited evidence base, further studies are essential to validate these results.

The results of pharmacoeconomic studies on emicizumab are affected by various factors such as the healthcare system, drug prices, patient preferences, values, economic levels, and burden thresholds in different countries or regions. In addition, the clinical practice guidelines, clinical data sources, and prescription preferences of physicians in different countries also have a significant impact on the results of the economic evaluation of emicizumab. In addition to the differences in target populations and medical insurance reimbursement policies in pharmacoeconomic evaluations, factors such as drug costs and healthcare costs varied across different studies. These differences are to some extent inevitable.

Some limitations were identified in this study. While the CHEERS 2022 checklist was employed to enhance the interpretability and decision-making utility of health economic evaluations, it solely focuses on report completeness and accuracy rather than the reporting process itself. For example, the cost-effectiveness analysis conducted by Kragh et al. (30) utilized UK list prices but lacked UK-specific patient data, relying instead on individualized prophylaxis arm data from the A-LONG study and its long-term extension ASPIRE study, which included only a small UK participant subset. Additionally, although fully declared, the authors’ financial ties to the comparator product’s manufacturer introduce potential conflict of interest that may influence result interpretation. Additionally, the assessment of reporting quality of studies is highly subjective, and the CHEERS 2022 checklist necessitates researchers to subjectively determine the compliance of literature entries. Consequently, differences in evaluators may lead to varying interpretations of each entry, resulting in differing evaluation outcomes. Furthermore, the CHEERS 2022 checklist is not intended as a scoring tool or a tool to assess the appropriateness of methods. This study has independently assigned scores to each item to quantitatively assess the reporting quality of each study. However, if scoring is required, the Quality of Health Economic Studies (QHES) scale (39) may offer a more structured alternative. To address these gaps, future research could adopt mixed-method designs integrating qualitative and quantitative approaches. Expanding use of pharmacoeconomic guidelines alongside CHEERS 2022 would also provide a more comprehensive evaluation framework. A further limitation is the restriction to English or Chinese studies, which may have excluded relevant pharmacoeconomic research from Latin America, Europe, and other regions. This could introduce bias by omitting region-specific evidence (e.g., local payer priorities, cost structures) that influences emicizumab’s cost-effectiveness modeling, limiting the generalizability of the conclusions. Future research should involve collaboration with multilingual researchers to incorporate studies published in additional languages (e.g., Spanish, French) to address this gap. Moreover, real-world evidence (RWE) is critical for augmenting clinical trial data, offering actionable insights into emicizumab’s real-world effectiveness and cost-effectiveness in diverse healthcare settings. Prioritizing RWE integration will strengthen economic evaluations and inform resource allocation decisions.

## 5 Conclusion

A systematic review of the 17 pharmacoeconomic research on prophylaxis emicizumab use for bleeding in people with HA was conducted, which revealed that the related studies published were generally of good reporting quality. Current studies revealed that emicizumab was more cost-effective compared to BPAs in people with HA with inhibitors, but less cost-effectiveness compared to rFVIIIFc for people with HA without inhibitors. Its cost-effectiveness compared to rFVIII varied across different countries. With advancing gene therapies, more studies comparing emicizumab with valoctocogene roxaparvovec are needed. Future research should also include accurate country-specific cost estimations and address identified quality gaps to strengthen evidence for HA clinical decision-making.

## Data Availability

The original contributions presented in the study are included in the article. Further inquiries can be directed to the corresponding author.

## Glossary

HA: hemophilia A
FVIII: factor VIII
WFH: World Federation of Hemophilia
EHL: extended half-life
BPAs: bypassing agents
aPCC: activated prothrombin complex concentrates
rFVIIa: recombinant activated factor VII
ISTH: International Society on Thrombosis and Haemostasis
FIXa: activated factor IXa
FX: factor X
ABR: annual bleeding rate
rFVIIIFc: recombinant factor VIII Fc fusion protein
AUD: Australian dollar
USD: United States dollar
SHL: standard half-life
QALYs: quality-adjusted life years
PRISMA: Preferred Reporting Items for Systematic Reviews and Meta-Analyses
PROSPERO: International Prospective Register of Systematic Reviews
NHSEED: National Health Service Economic Evaluation Database
HTA: Health Technology Assessment
CNKI: China National Knowledge Infrastructure
VIP: VIP China Science and Technology Journal database
CHEERS: Consolidated Health Economic Evaluation Reporting Standards
ISPOR: International Society for Pharmacoeconomics and Outcomes Research
DRM: dominance ranking matrix
rFVIII: recombinant factor VIII
ITI: immune tolerance induction
NHS: National Health Service
Euro: European dollar
ICER: incremental cost-effectiveness ratio
GDP: gross domestic product
US: the United States
the ICER, US research group: the Institute for Clinical and Economic Review
ODT: on-demand therapy
LDP: low-dose prophylaxis
IDP: intermediate-dose prophylaxis
HDP: high-dose prophylaxis
INR: Indian rupees
UK: the United Kingdom
GBP: Great Britain pound
FDA: Food and Drug Administration
MVBP: maximum value-based price
CTAF: California Technology Assessment Forum
QHES: Quality of Health Economic Studies
RWE: real-world evidence
